# Skeletal muscle mitochondrial dysfunction in contemporary antiretroviral therapy: a single cell analysis

**DOI:** 10.1097/QAD.0000000000003334

**Published:** 2022-07-15

**Authors:** Matthew Hunt, Megan M Mcniff, Amy E Vincent, Caroline Sabin, Alan Winston, Brendan Ai Payne

**Affiliations:** 1 Wellcome Centre for Mitochondrial Research, Translational and Clinical Research Institute, Newcastle University, Newcastle-upon-Tyne, UK; 2 Dermatology and Venereology Division, Department of Medicine (Solna), Karolinska Institutet, Stockholm, Sweden; 3 Centre for Clinical Research, Epidemiology, Modelling and Evaluation, Institute for Global Health, University College London, London, UK; 4 Division of Medicine, Imperial College London, St Mary’s Campus, London, UK; 5 Department of Infection and Tropical Medicine, Newcastle-upon-Tyne Hospitals NHS Foundation Trust, Newcastle-upon-Tyne, UK

**Keywords:** Mitochondria, HIV, anti-retroviral therapy, mtDNA deletion, skeletal muscle, single cell analysis

## Abstract

**Objective:**

To quantify mitochondrial function in skeletal muscle of people treated with contemporary anti-retroviral therapy.

**Design:**

Cross-sectional observational study.

**Methods:**

Quantitative multiplex immunofluorescence was performed to determine mitochondrial mass and respiratory chain complex abundance in individual myofibres from tibialis anterior biopsies. Individual myofibres were captured by laser microdissection and mitochondrial DNA (mtDNA) content and large-scale deletions were measured by real-time PCR.

**Results:**

45 anti-retroviral therapy (ART) treated people with HIV (PWH, mean age 58 years, mean duration of ART 125 months) were compared with 15 HIV negative age-matched controls. Mitochondrial complex I (CI) deficiency was observed at higher proportional levels in PWH than negative controls (p 0.008). Myofibre mitochondrial mass did not differ by HIV status.

No ART class was significantly associated with mitochondrial deficiency, including prior exposure to historical NRTIs (nucleoside analogue reverse transcriptase inhibitors) associated with systemic mitochondrial toxicity.

To exclude an effect of untreated HIV, we also studied skeletal muscle from 13 ART-naïve PWH (mean age 37). These showed negligible CI defects, as well as comparable myofibre mitochondrial mass to ART-treated PWH.

Most CI-deficient myofibres contained mtDNA deletions. No mtDNA depletion was detected.

**Conclusion:**

Here we show that PWH treated with contemporary ART have mitochondrial dysfunction in skeletal muscle, exceeding that expected due to age alone. Surprisingly, this was not mediated by prior exposure to mitochondrially toxic NRTIs, suggesting novel mechanisms of mitochondrial dysfunction in contemporary ART-treated PWH. These findings are relevant for better understanding successful ageing in PWH.

## Background

Despite successful viral suppression through anti-retroviral therapy (ART), some people with HIV (PWH) show an excess of adverse ageing phenotypes such as frailty and sarcopenia, as well as reduced physical function [1, 2]. These phenotypes are predictive of incident morbidity and mortality [3]. Maintenance of skeletal muscle quantity and quality is an important predictor of healthy ageing in the general population, but skeletal muscle has been little studied in people ageing with HIV.

Mitochondrial dysfunction is widely considered as being a causative biological factor in human ageing [4]. Indeed, recent data from the general population suggest that skeletal muscle mitochondrial dysfunction is an important mediator of frailty, but comparable studies in PWH are lacking [5]. Exposure to some historical nucleoside analogue reverse transcriptase inhibitors (NRTIs) such as zidovudine (AZT), zalcitabine (ddC), stavudine (d4T) and didanosine (ddI) has been linked to mitochondrial abnormalities in several tissues, including skeletal muscle [6–8]. These NRTIs disrupt mitochondrial DNA (mtDNA) replication, leading to a reduction in cellular mtDNA content (depletion) and the accumulation of mtDNA mutations [9]. In contrast, contemporary ART is generally considered relatively free from mitochondrial toxicity, in part because newer NRTIs have a low binding affinity to the mitochondrial polymerase, pol-γ [10, 11]. Other classes of ART do not affect the function of pol-γ, but have nevertheless been hypothesized to cause mitochondrial dysfunction. Whilst human data are limited, various study modalities have suggested that mitochondrial dysfunction may occur with newer NRTIs, protease inhibitors (PIs) and non-nucleoside reverse transcriptase inhibitors (NNRTIs) [12–16].

Published data indicate an excess of mitochondrial dysfunction in skeletal muscle of younger PWH, aged ≤50 years [6]. In this age group, a negligible effect of age on mitochondrial function is expected [17]. In these younger PWH, mitochondrial dysfunction was strongly predicted by prior exposure to those historical NRTIs associated with systemic mitochondrial toxicity. It remains unknown how these findings will translate to older PWH. Will a legacy effect of prior NRTI exposure dominate over age-associated mitochondrial dysfunction? Alternatively, will other factors drive mitochondrial dysfunction in older PWH? Given the importance of skeletal muscle function as a mediator of ageing phenotypes in the general population, our aims were to objectively quantify mitochondrial function at the cellular level in skeletal muscle in the setting of contemporary ART, and to determine the relative contributions of ageing, HIV, and ART.

## Methods

### Participants

The study was approved by local research ethics committee (17/NE/0015). All participants had given prior written informed consent for collection and retention of tissue for research purposes. Participants with other neuromuscular disease were excluded. Clinical and HIV characteristics were captured at the time of study visits and by case note review. We specifically recorded both current and prior ART history, including prior exposure to those historical NRTIs with systemic mitochondrial toxicity (AZT, d4T, ddI, ddC). As age is known to be a strong predictor of mitochondrial dysfunction, HIV negative controls were carefully age-matched to the ART-treated PWH. Some ART-treated PWH and all HIV negative controls were recruited as part of other studies of HIV and ageing [18]. In addition, archival samples were obtained from the Newcastle Academic Health Partners Biobank.

In addition, we wished to determine whether untreated HIV infection was associated with mitochondrial defects in skeletal muscle. As almost all PWH in current clinical care are on ART, we obtained archival samples from ART-naïve PWH. By necessity, these were not age-matched. As such, data were not compared statistically to the ART-treated group but are provided for context.

In order to calibrate the multiplex immunofluorescence staining, additional skeletal muscle was obtained from the distal hamstring of 5 young adults (aged 18 to 30 years) without other medical conditions who were undergoing anterior cruciate ligament repair surgery (here termed ‘calibrator’ samples).

### Multiplex immunofluorescence of skeletal muscle

Muscle samples were obtained by percutaneous biopsy of tibialis anterior under local anaesthesia and snap-frozen in the liquid phase of isopentane, cooled in liquid nitrogen. Muscle cryosections (10μm) were subjected to a validated automated multiplex immunofluorescence assay, previously developed within our group [19]. Briefly, this technique simultaneously labels for four myofibre components, as follows. Laminin labels the myofibre boundary. VDAC1 (also known as porin) is an abundant mitochondrial outer membrane protein. As such, it provides a measure of the total mitochondrial mass within a myofibre. The other two targets labelled were subunits of the mitochondrial respiratory chain complexes I (CI, subunit NDUFB8) and IV (CIV, subunit MTCO1). Mitochondrial respiratory chain complex deficiency in skeletal muscle is predominantly seen in CI and CIV. For each batch of skeletal muscle analysed, additional cryosections were stained in parallel using only the primary antibody for laminin, along with all the secondary antibodies (termed ‘noprimary controls’, NPC). In order to minimize batch-effects, all batches of cryosections analysed comprised a mixture of all experimental groups, NPC and calibrator samples.

### Image analysis

Images were tiled in Zen 2011 Blue Edition (Zeiss), then automatically segmented using the laminin signal (405 nm) into individual myofibres. Correct segmentation was confirmed by manual inspection.

The average fluorescence intensity in the respective channels for NDUFB8 (647 nm), MTCO1 (488 nm) and VDAC1 (546 nm) for each individual myofibre was then automatically quantified using in-house software (in MatLab 2015a). The mean optical density signals for the VDAC1, NDUFB8 and MTCO1 channels from the NPC were used to correct the signals from the experimental samples for background fluorescence [20]. NDUFB8 and MTCO1 abundance in individual myofibres was then expressed relative to VDAC1 abundance for the same myofibre. This normalises mitochondrial respiratory chain protein abundance for overall mitochondrial mass within the myofibre.

The abundance of VDAC1, NDUFB8 and MTCO1 within individual myofibres from the calibrator samples (young healthy persons) was then used to derive the expected distribution for these parameters. Using an in-house R Shiny script, values for individual myofibres from the experimental samples were then expressed relative to this expected distribution as z-scores (that is, an expected mean z-score of 0 and standard deviation of 1). Myofibres with NDUFB8 or MTCO1 z-score <-6 were classified as ‘deficient’, -3 to -6 as ‘intermediate’, and >-3 as ‘normal’.

### Single cell molecular analyses

We then determined the molecular basis of observed mitochondrial CI defects at the single cell level. Serial cryo-sections (15μm) were placed on PEN membrane slides (Zeiss). CI deficient, intermediate, and normal myofibres were captured by laser microdissection into individual 0.2ml tubes containing 15μL single-cell lysis buffer (0.5M Tris-HCl, 0.5% Tween 20, 1% Proteinase K, pH 8.5) using a PALM system (Zeiss), and incubated at 55°C for 3 hours followed by heat inactivation for 10 minutes at 95°C.

Quantitative real-time PCR (qPCR) was performed on a CFX96 Touch Real-Time PCR Detection System (Bio-Rad) using a multiplex assay targeting the mtDNA-encoded genes *MT-ND1* (forward primer L3485-3504; reverse primer H3532-3553; probe L3506-3529) and *MT-ND4* (forward primer L12087-12109; reverse primer H12140-12170; probe L12111-12138), as described previously [21]. *MT-ND1* is located in the minor arc of the mtDNA genome and *MT-ND4* in the major arc. Targets in *MT-ND1* and *MT-ND4* are commonly used to screen for large-scale mtDNA deletions by qPCR as the majority of large-scale mtDNA deletions affect the major arc and thus remove *MT-ND4* but retain *MT-ND1.* 2μL of DNA lysate from individual myofibres were amplified in triplicate. Mastermix comprised: iTaq (Bio-Rad); 75nM of each primer; 200nM of each probe. Amplification conditions were: 3 minutes at 95°C, then 39 cycles of 10 seconds at 95°C followed by 1 minute at 62°C. DNA extract from whole blood of a healthy individual was also included on each qPCR run as a control sample as this contains negligible levels of mtDNA deletions.

We screened for mtDNA deletions in individual myofibres by comparing threshold cycle (C_t_) values of *MT-ND1* to *MT-ND4* relative to those C_t_ values for the control sample. That is, δδC_t_ = [C_t_(*MT-ND1*)_sample_ - C_t_(*MT-ND4*)_sample_] – [C_t_(*MT-ND1*)_control_ - C_t_(*MT-ND4*)_control_]. We screened for mtDNA depletion (reduction in overall cellular mtDNA content) in individual CI-deficient myofibres by considering the calculated starting quantity (SQ) of mtDNA relative to the 5^th^ centile of SQ in CI-normal myofibres from the same individuals.

### Statistical analyses

Statistical analyses were performed in SPSS v24 (IBM) and Prism v9.3.1 (Graphpad). Differences in the proportion of myofibres with CI and CIV deficiency was compared between ART exposure groups using Mann-Whitney tests and correlation between CI deficiency and continuous variables by Spearman rank. Statistical significance was set at p < 0.05.

## Results

### Cohort characteristics

We investigated skeletal muscle biopsies from 45 ART-treated PWH. Mean age was 57.6 years (SD 7.5) and well-matched to the HIV negative control group (mean age 59.7, SD 7.3). 93% were male and 98% were of white European ethnicity (Table 1). Mean duration of diagnosed HIV infection was 191 (SD 89) months and mean duration of ART was 125 (66) months. Half (51%) had history of prior exposure to NRTIs with systemic mitochondrial toxicity. All subjects had HIV-1 RNA plasma viral load (VL) <400 copies/mL and 81% had VL <50 copies/mL. Mean CD4 count was 619 (207) cells/μL and all subjects had CD4 count >200 cells/μL. Amongst the ART-naïve PWH, mean age was 36.8 years (SD 10.1), mean duration of diagnosed HIV 74 (56) months, and mean CD4 count 635 (414) cells/μL.

### Mitochondrial respiratory chain complex deficiency in individual myofibres

**Figure 1:**
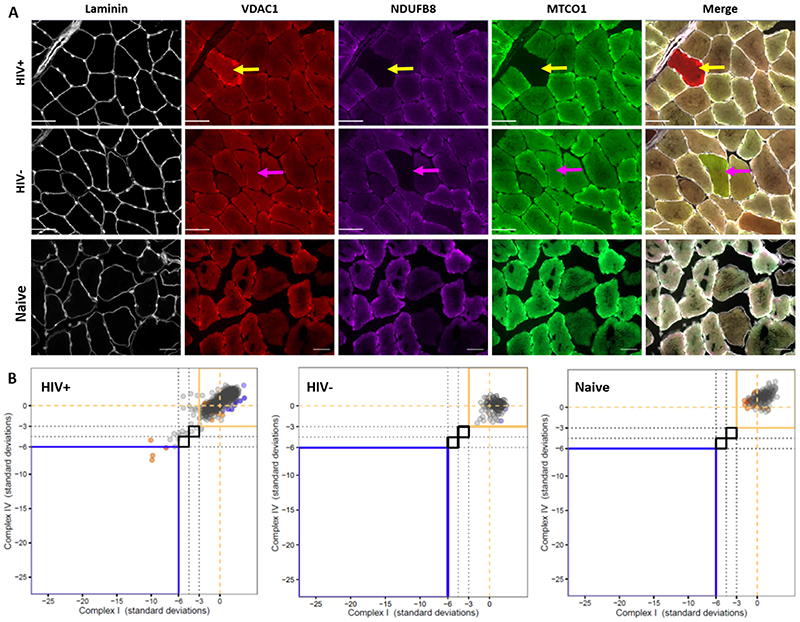
Quantitative multiplex immunofluorescence analysis of mitochondrial dysfunction in individual myofibres. **(A)** Representative examples of multiplex immunofluorescence for mitochondrial respiratory chain complex 1 (CI, NDUFB8), complex 4, (CIV, MTCO1), mitochondrial mass (VDAC1) and laminin in an ART-treated PWH, an HIV negative control, and an ART-naïve PWH. The yellow arrow indicates a myofibre deficient in CI and CIV, with hyperintensity of VDAC1, indicating compensatory mitochondrial proliferation. The pink arrow indicates a myofibre deficient in CI but not CIV. Scale bars are 100μm. **(B)** Example 2D dot plots of CI z-scores (x-axis) and CIV z-scores (y-axis) of individual myofibres from two ART-treated PWH. Dotted lines are at z-score of 0, i.e. the expected mean. Box outlined in upper right quadrant of each panel contains myofibres with normal CI and CIV abundance. Each dot represents an individual myofibre and is coloured based on VDAC1 intensity (mitochondrial mass) category: light blue = ‘low’ (z -3 to -2), cream = ‘normal’ (z -2 to +2), orange = ‘high’ (z +2 to +3), and red = ‘very high’ (z > +3).

Representative immunofluorescence images of mitochondrial mass and respiratory chain complex abundance in individual myofibres are shown (Figure 1A). We observed a mosaic pattern of individual myofibres which were deficient in CI with or without CIV deficiency in ART-treated PWH. Individual myofibres from ART-treated PWH showed a range of CI deficiency, and to a lesser extent, CIV deficiency (illustrative cases shown in Figure 1B).

**Figure 2:**
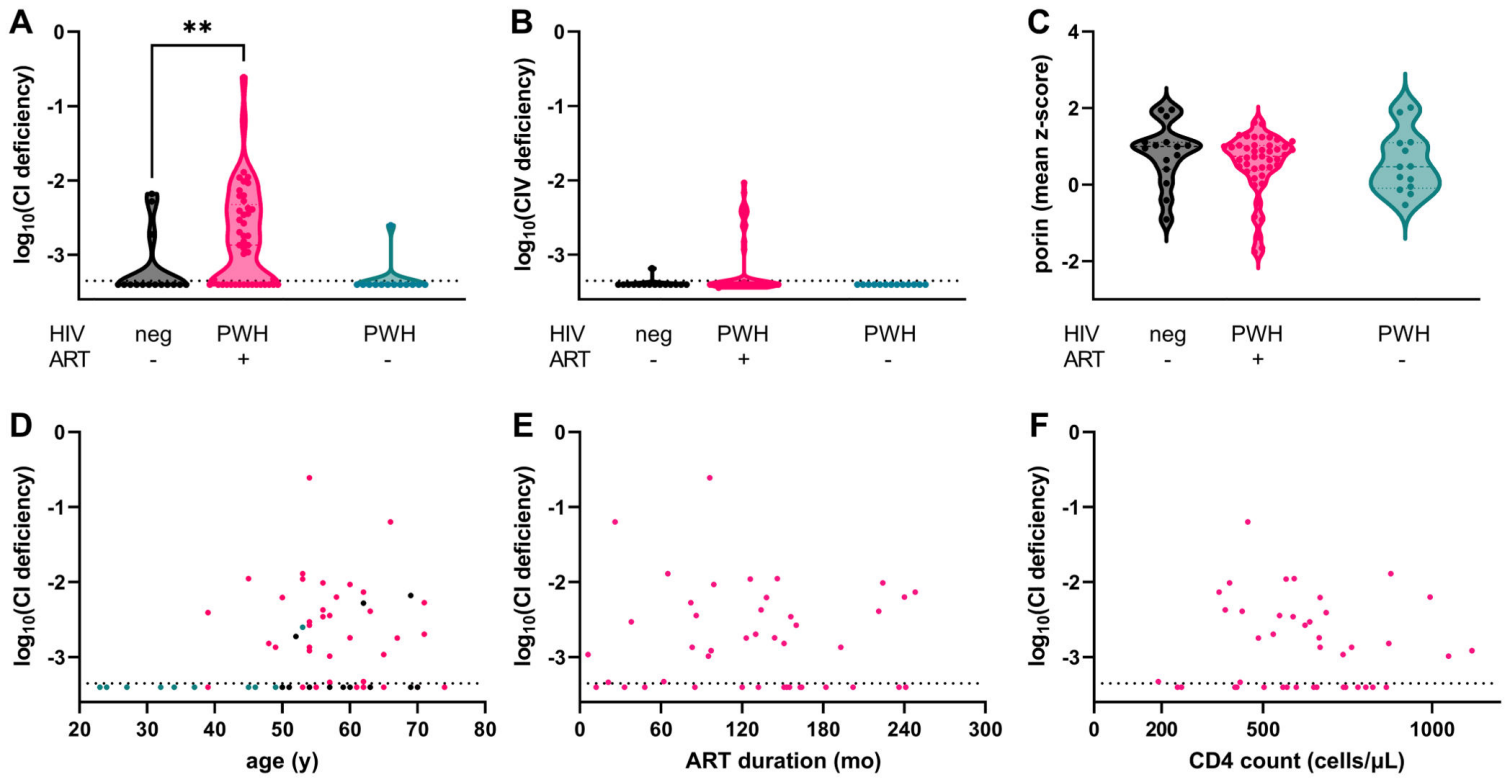
Mitochondrial respiratory chain deficiency in single skeletal myofibres. Mitochondrial complex I (CI) (**A**) and complex IV (CIV) (**B**) and mass (**C**) according to HIV and ART status (x-axes). CI and CIV expressed as proportion of myofibres showing respiratory chain deficiency. Mitochondrial mass (porin / VDAC1) expressed as mean z-score. Mitochondrial CI deficiency by age (**D**), ART duration (**E**), and current CD4 cell count (**F**). ** p <0.01; dots represent individual subjects; dotted line on y-axis, lower limit of detection of assay.

We compared the proportions of CI and CIV-deficient myofibres between ART treatment groups. ART-treated PWH showed significantly higher proportional levels of CI-deficient myofibres than age-matched controls (p 0.008), but this difference did not reach significance for CIV-deficient myofibres (p 0.11, Figure 2A-B). Levels of CI and CIV deficiency in the ART-naïve PWH were negligible. As myofibres with reduced CI abundance were more common than myofibres with reduced CIV abundance, we focused our subsequent analyses on CI abnormalities.

We assessed overall mitochondrial mass in individual myofibres by considering the VDAC1 intensity. There was no significant difference in median myofibre mitochondrial mass between ART-treated PWH and age-matched controls (p 0.23, Figure 2C). Mitochondrial mass in ART-naïve PWH was also similar.

### Clinical and HIV-related predictors of mitochondrial dysfunction

None of age, current CD4 count, duration since HIV diagnosis, or duration on ART predicted proportional CI defect (age, Spearman rho -0.16, p 0.31; CD4 count, rho -0.07, p 0.7; HIV duration, rho -0.23, p 0.14; ART duration, rho -0.01, p 0.9; Figure 2D-F).

### ART class and mitochondrial dysfunction

**Figure 3:**
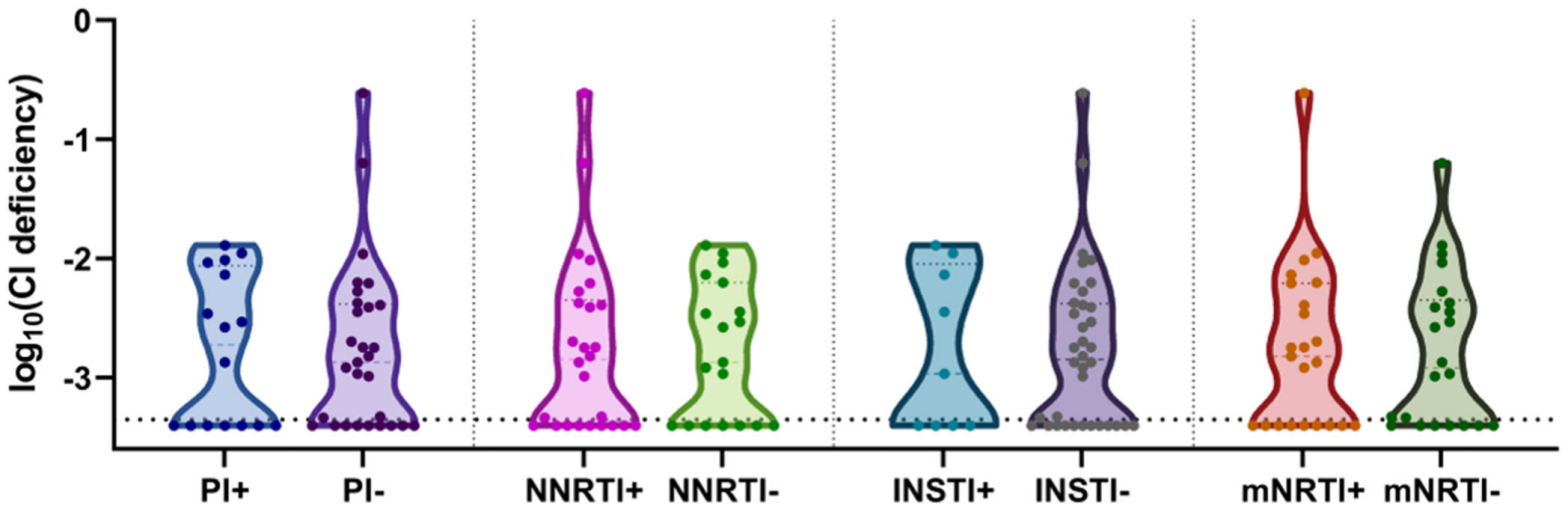
Skeletal muscle mitochondrial respiratory chain deficiency by ART class exposure. Mitochondrial complex 1 (CI) deficiency in ART-treated PWH. PI, protease inhibitor (current exposure); NNRTI, non-nucleoside reverse transcriptase inhibitor (current exposure); INSTI, integrase inhibitor (current exposure); mNRTI, nucleoside reverse transcriptase inhibitor with systemic mitochondrial toxicity (prior exposure). Dotted line on y-axis is limit of detection of assay. Dots are individual subjects.

We next examined whether prior exposure to NRTIs with systemic mitochondrial toxicity (AZT, d4T, ddC, ddI) was associated with mitochondrial dysfunction in skeletal muscle. Surprisingly, there was no difference in proportional levels of CI deficient myofibres by prior NRTI exposure (p 0.9, Figure 3).

We then investigated whether there was evidence of skeletal muscle mitochondrial dysfunction in PWH currently treated with ART from classes other than NRTIs. Here we saw no association between proportional level of CI deficiency and any ART class (PI, p 0.9; NNRTI, p 0.9; integrase inhibitor (INSTI), p 1.0, Figure 3).

### Molecular basis of CI-deficiency in single myofibres

Finally, we explored the molecular basis of the observed mitochondrial defects by subjecting single myofibres (n = 90) to qPCR analyses. Specifically, CI-deficient (n = 24), CI-intermediate (n = 27), and CI-normal (n = 39) myofibres were isolated by laser capture microdissection from ART-treated PWH. We included participants both with (n = 5) and without (n = 4) prior exposure to historical NRTIs with systemic mitochondrial toxicity.

**Figure 4:**
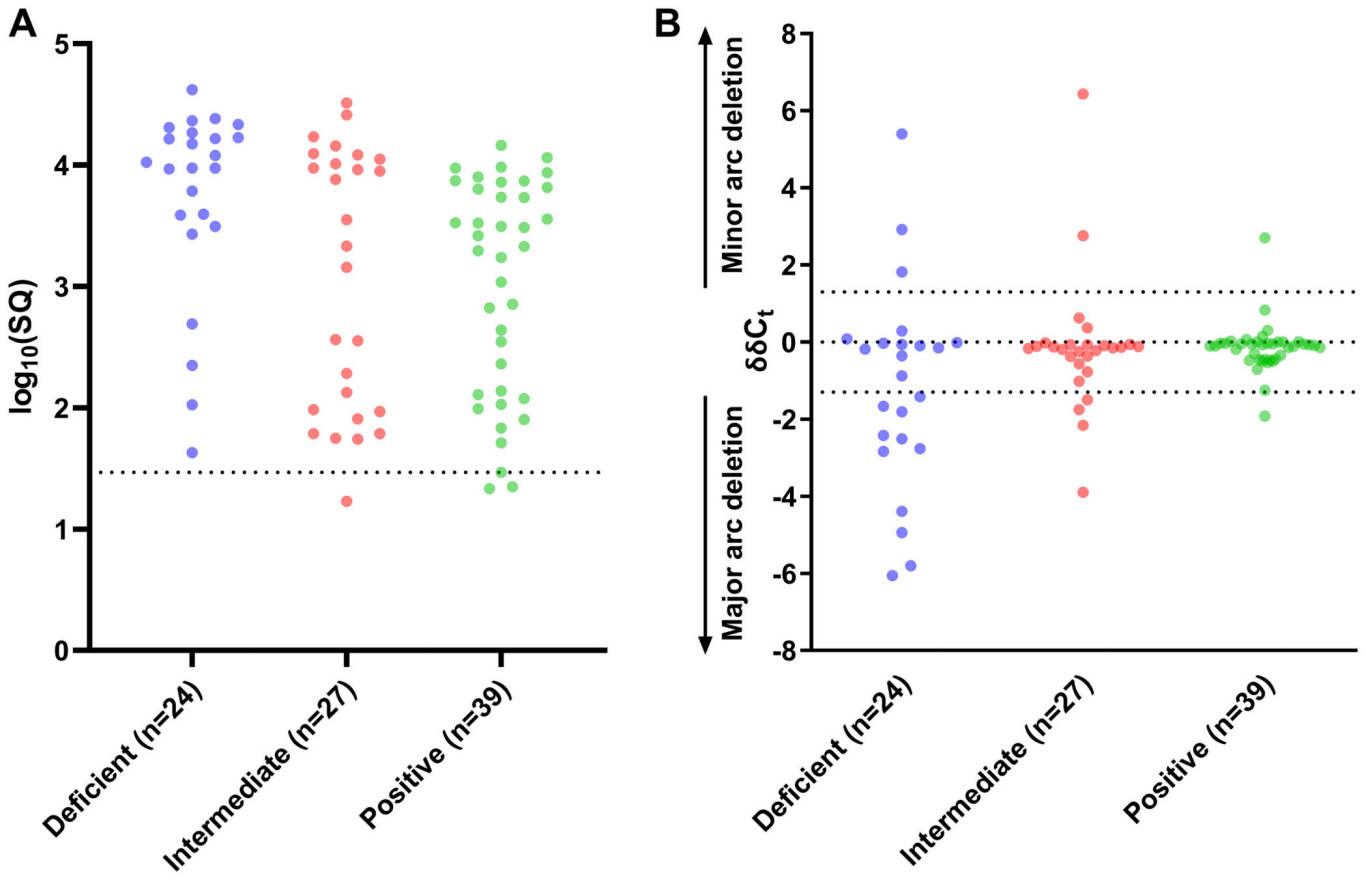
mtDNA analysis of single myofibres. qPCR analysis of individual CI-deficient, CI-intermediate and CI-positive myofibres from ART-treated PWH. Each dot represents an individual myofibre. **(A).** mtDNA content (SQ, arbitrary units). Dotted line is 5^th^ centile of SQ for CI-positive myofibres. **(B).** Large-scale mtDNA deletions. Mutation loads are expressed as δδC_t_ (difference in *MT-ND1* and *MT-ND4* C_t_ (cycle threshold) values relative to undeleted assay control). Deletions in the major arc of the mtDNA genome delete *MT-ND4* and are shown as negative δδC_t_ values. Minor arc deletions delete *MT-ND1* and are shown as positive δδC_t_ values. Thin dotted lines show 2 standard deviations from the mean for CI-positive myofibres.

Firstly, we excluded mtDNA depletion as a cause of CI deficiency in myofibres. In keeping with our observations of cellular mitochondrial mass, we saw no evidence of reduced mtDNA content in individual CI-deficient myofibres (Figure 4A). We then investigated the presence of large-scale mtDNA deletions. 14 (58%) CI-deficient myofibres contained detectable large-scale mtDNA deletions (Figure 4B). Altogether, 74% of mtDNA deletions occurred in the major arc of the mtDNA genome, whilst 26% occurred in the minor arc. We found no significant difference in the prevalence of myofibres with mtDNA deletions according to history of exposure to historical NRTIs with systemic mitochondrial toxicity (contemporary NRTI exposure only, n = 11, 24%; historical NRTI exposure, n = 12, 27%; p 0.81).

## Discussion

Here we demonstrate defects of skeletal muscle mitochondrial function at the single cell level in PWH who have only ever been exposed to contemporary ART, despite this being regarded as largely free from mitochondrial toxicity. As mitochondrial dysfunction is known to increase with age [22, 23], we carefully controlled for this effect by age-matching the HIV negative control group. We can therefore conclude that mitochondrial dysfunction exceeds that expected for age in older ART-treated PWH.

These novel observations raise several mechanistic questions. As expected, we observed that the defects of respiratory chain complexes seen in single myofibres were explained mostly by mtDNA large-scale deletions. The pattern of molecular defects did not differ according to historical NRTI exposure. This is surprising as previous *in vitro* data suggest that contemporary ART does not inhibit the replication of mtDNA [9]. Nevertheless, it is conceivable that very prolonged exposure to a contemporary NRTI *in vivo* could be sufficient to promote mtDNA deletions [13]. Another possibility is that other ART classes might contribute to mitochondrial dysfunction in contemporary ART. For example, limited *in vitro* data suggest that the NNRTI efavirenz may impair mitochondrial function [16, 24]. We therefore examined for an effect of 3^rd^ agent (NNRTI / PI / INSTI) exposure in our dataset, but this was not seen. Furthermore, the lack of any mitochondrial defects in the treatment-naïve group argues against a major effect of HIV itself. However, by necessity, this group was of younger age and had shorter duration of HIV infection than the ART-treated group. Overall, it seems most likely that that the observed skeletal muscle mitochondrial dysfunction in ART-treated PWH is multi-factorial, perhaps driven by factors known to be present even with suppressive ART, such as chronic inflammation or oxidative stress [25, 26].

A key strength of our study was that we have employed techniques that can objectively quantify mitochondrial deficiency with single-cell resolution. Given the stochastic nature of somatic (acquired) mtDNA defects within post-mitotic tissues such as skeletal muscle, studies of homogenized tissue may miss these defects [27]. Unlike the traditional cytochrome c oxidase histochemical technique which detects only CIV defects, our immunofluorescence assay also allows for the detection of CI defects. This is highly desirable as genes encoding CI subunits make up the greatest proportion of the mtDNA genome and are therefore most observed in association with mtDNA deletions. Our observation that CI defects predominate could also be of potential relevance to therapeutic interventions [28, 29]. As CI is a very large respiratory chain complex, comprised of multiple subunits (both mtDNA and nuclear DNA encoded), further work should examine whether there is also a reduction in fully-assembled CI, for example by BN-PAGE (blue native polyacrylamide gel electrophoresis).

These data underline the potential importance of skeletal muscle as an organ, and mitochondrial dysfunction as a mechanism, mediating adverse ageing phenotypes in PWH. Mechanistic studies such as ours may help lead to rational interventions for improving healthspan of older PWH.

## Supplementary Material

Supplemental Data File (.doc, .tif, pdf, etc.)

Response to reviewers

## Figures and Tables

**Table 1:** Cohort clinical and HIV-related characteristics. Current ART treatments: PI, protease inhibitor; NNRTI, non-nucleoside reverse transcriptase inhibitor; INSTI, integrase inhibitor. Prior mitochondrially-toxic NRTI exposure: zidovudine, stavudine, zalcitabine, didanosine.

	HIV negative	PWH	PWH
		ART-treated	ART-naive
n	15	45	13
**Age, mean (SD), years**	59.7 (7.3)	57.6 (7.5)	36.8 (10.1)
**Duration of diagnosed HIV, mean (SD), months**	N/A	191 (89)	74 (56)
**Duration on ART, mean (SD), months**	N/A	125 (66)	N/A
**Current CD4 count, mean (SD), cells/μl**	N/A	619 (207)	635 (414)
**PI treated, n (%)**	N/A	16 (36%)	N/A
**NNRTI treated, n (%)**	N/A	26 (58%)	N/A
**INSTI treated, n (%)**	N/A	9 (20%)	N/A
**Prior mitochondrial NRTI exposed, n (%)**	N/A	23 (51%)	N/A
