# Supplementary material for: Skeletal muscle mitochondrial dysfunction in contemporary antiretroviral therapy: a single cell analysis
Source: AIDS. Author manuscript; Available in PMC 2022 Nov 15. (PMC7613767; doi:10.1097/QAD.0000000000003334)
Supplement: Supplemental Data File (.doc, .tif, pdf, etc.) [file EMS150034-supplement-Supplemental_Data_File___doc___tif__pdf__etc__.pdf]

Skeletal muscle mitochondrial dysfunction in contemporary antiretroviral therapy: a single cell analysis.

Matthew HUNT, Megan M MCNIFF, Amy E VINCENT, Caroline SABIN, Alan WINSTON, Brendan AI PAYNE.

Supplementary material

Two-dimensional plots of mitochondrial respiratory chain complex abundance in individual myofibres from ART-treated PWH, age-matched HIV negative controls and ART-naïve PWH.

Each panel shows an individual participant (ART-treated PWH, n 45; HIV negative, 15; ART-naïve PWH, 13). Each dot is an individual myofibre (mean 850 myofibres analysed per sample).

X-axis is complex 1 (CI, NDUFB8), y-axis is complex 4 (CIV, MTCO1). Scale is abundance of CI / CIV expressed as a z-score (standard deviations) relative to calibrator samples from young healthy persons aged 18-30 years. Raw CI / CIV signal is background corrected to a no primary control and then expressed relative to mitochondrial mass (VDAC1 / porin) for that myofibre.

Upper right quadrant (bounded by thick orange lines) shows myofibres with normal CI and CIV abundance ($z > -3$). Lower left quadrant (bounded by thick blue lines) shows myofibres deficient in both CI and CIV ($z < -6$). Upper left quadrant shows myofibres deficient in CI only. Lower right quadrant shows myofibres deficient in CIV only. Myofibres with z-score between -6 and -3 have an intermediate level of CI / CIV abundance.

Individual myofibres are coloured according to mitochondrial mass (abundance of VDAC1 / porin) with scale as indicated below each panel.

ART-treated PWH

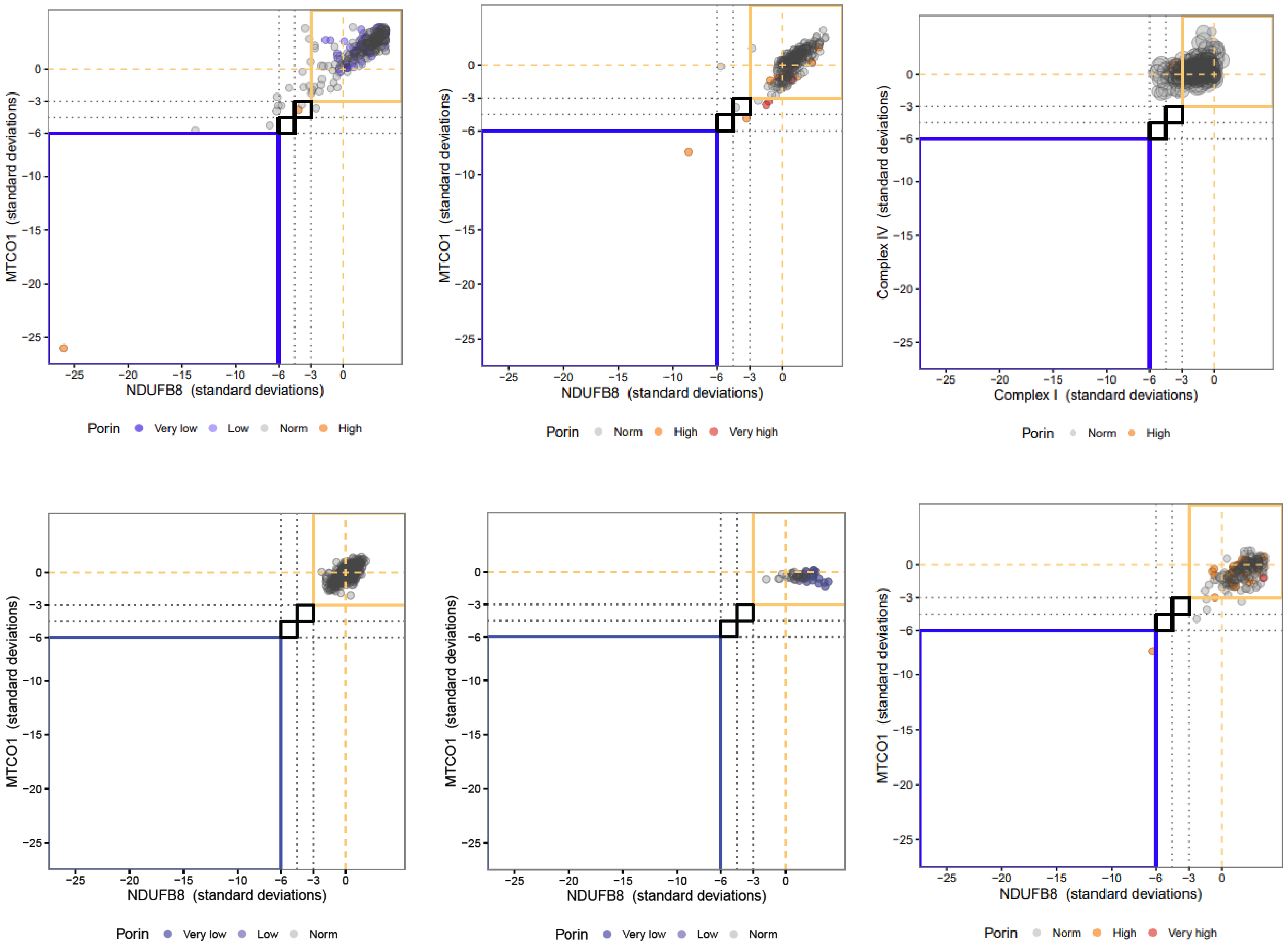

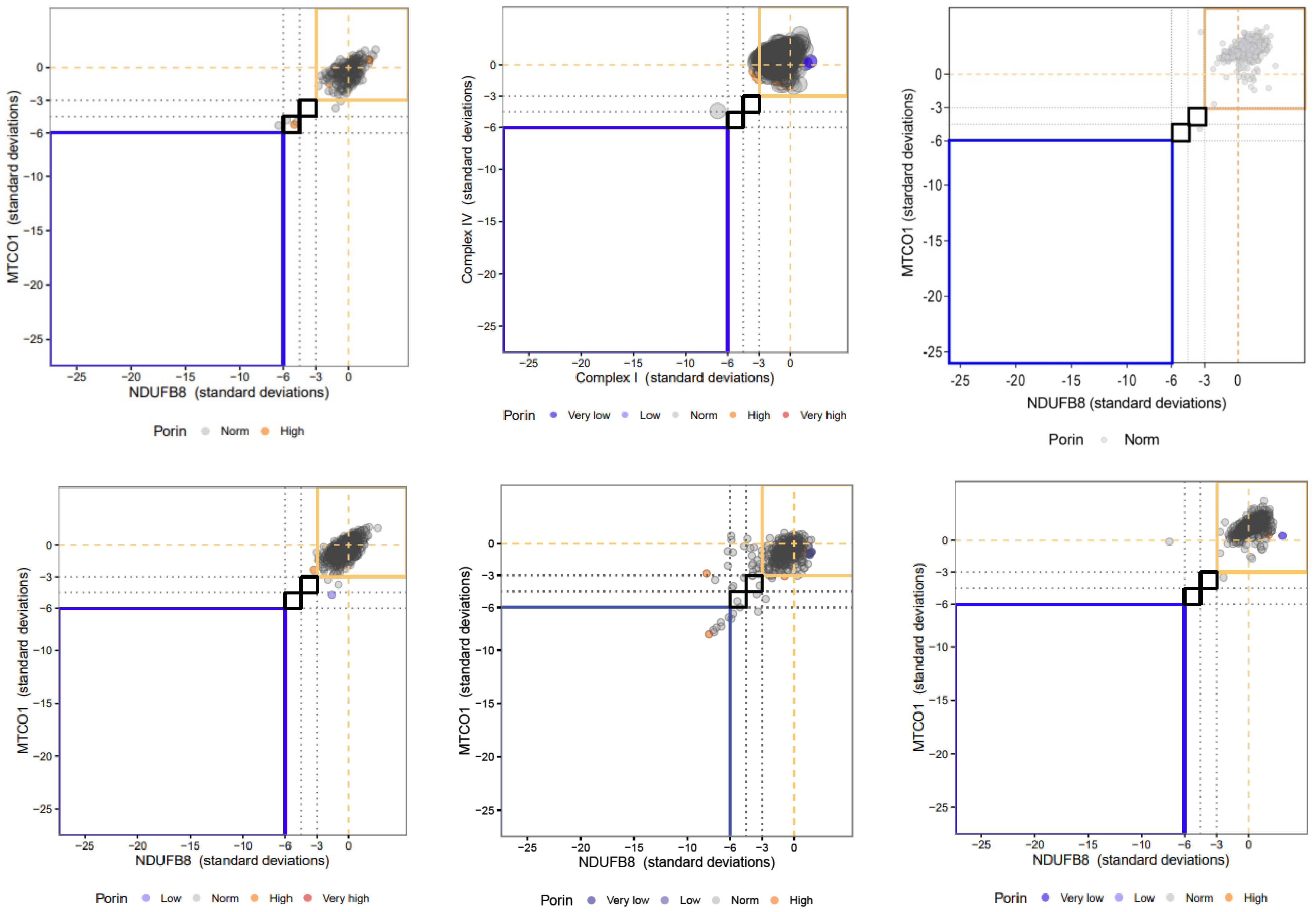

ART-treated PWH

ART-treated PWH

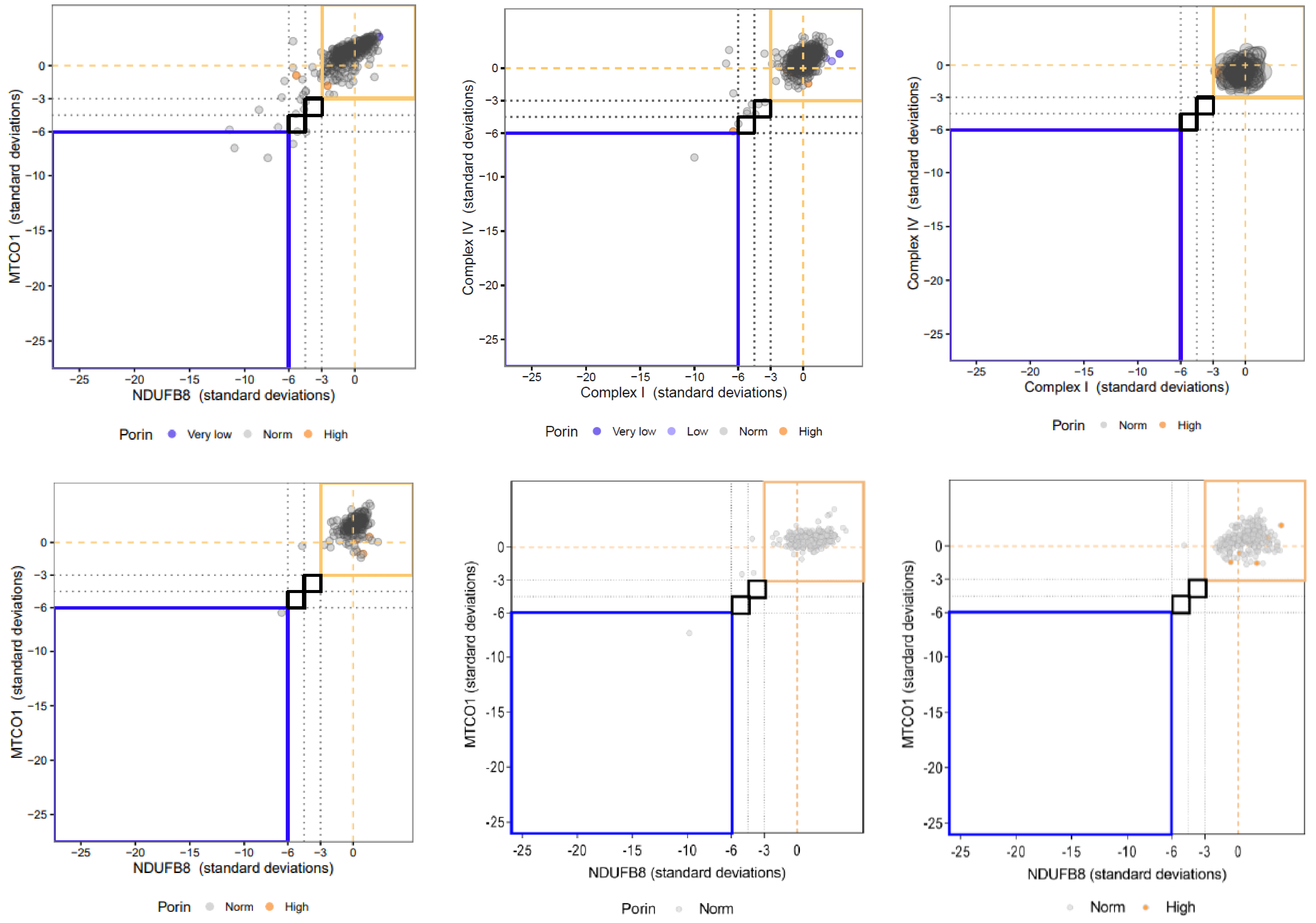

ART-treated PWH

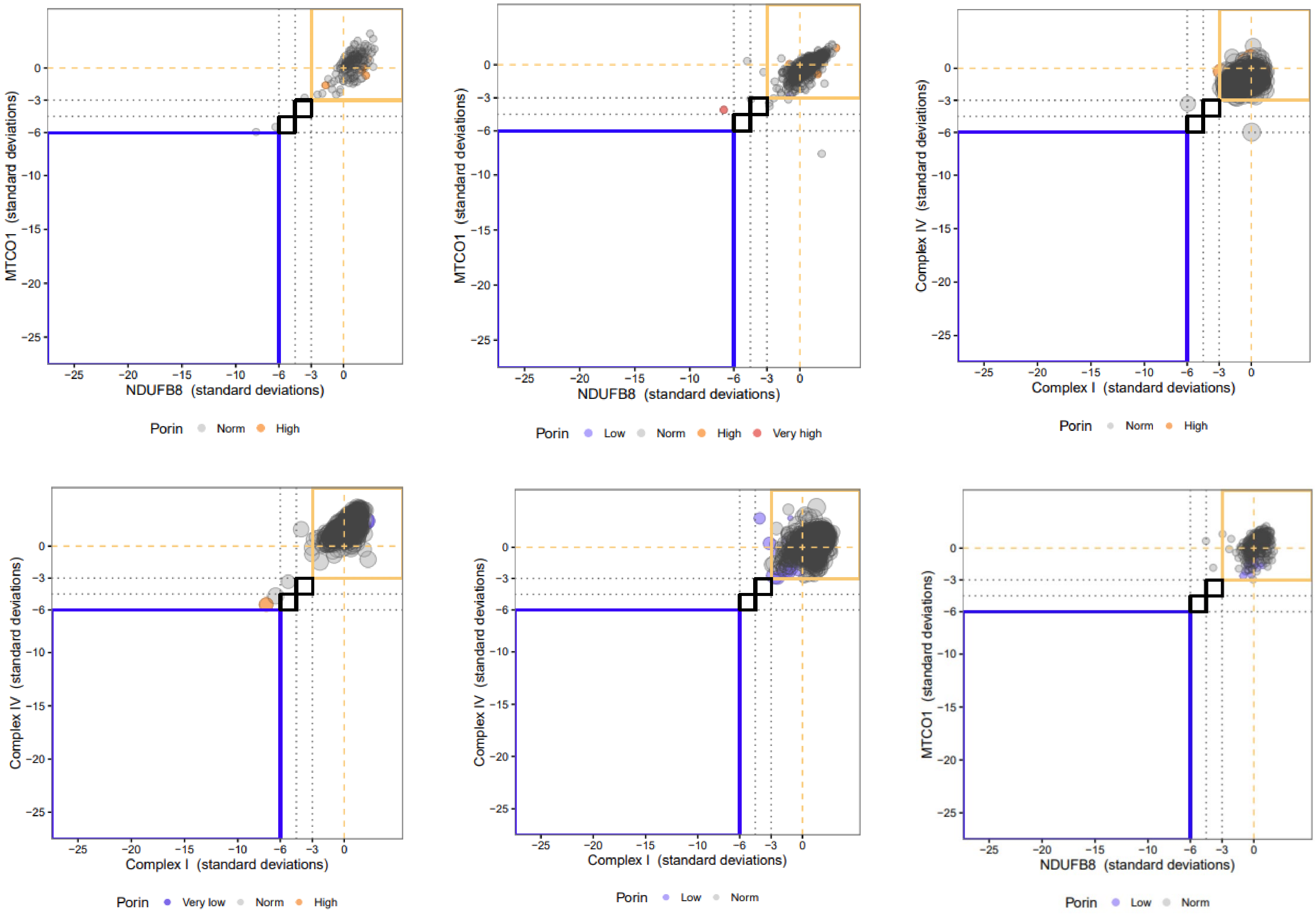

ART-treated PWH

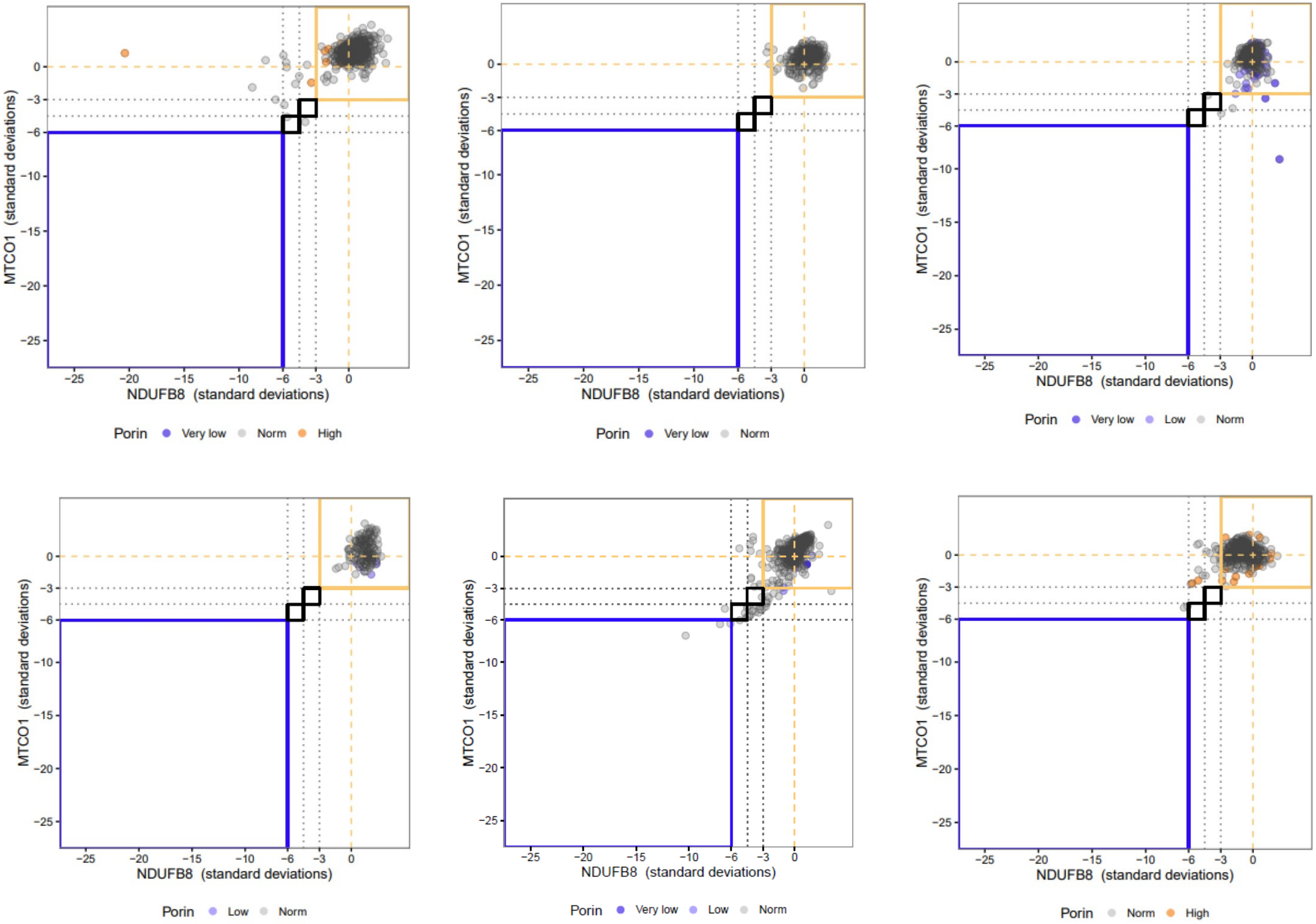

ART-treated PWH

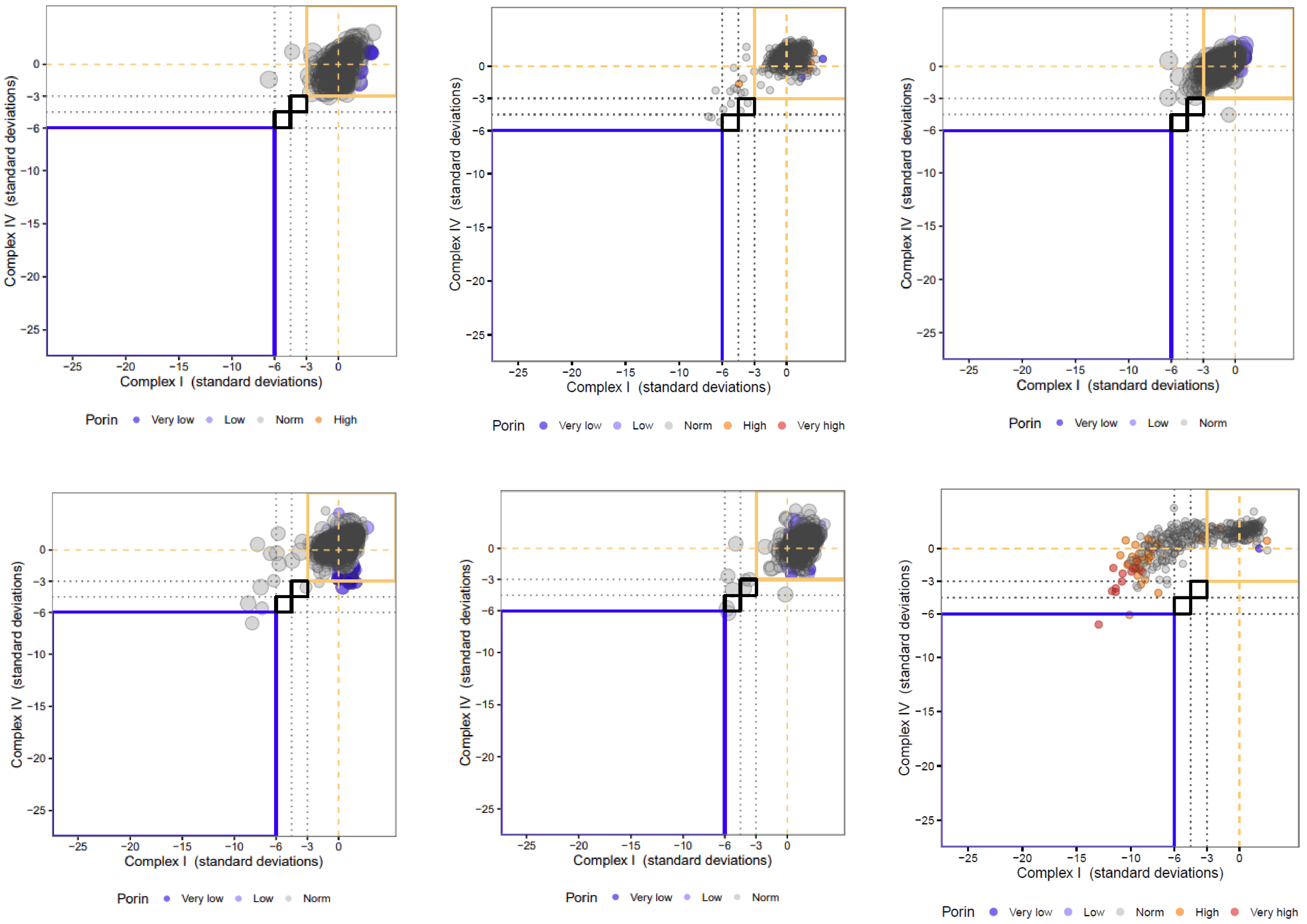

ART-treated PWH

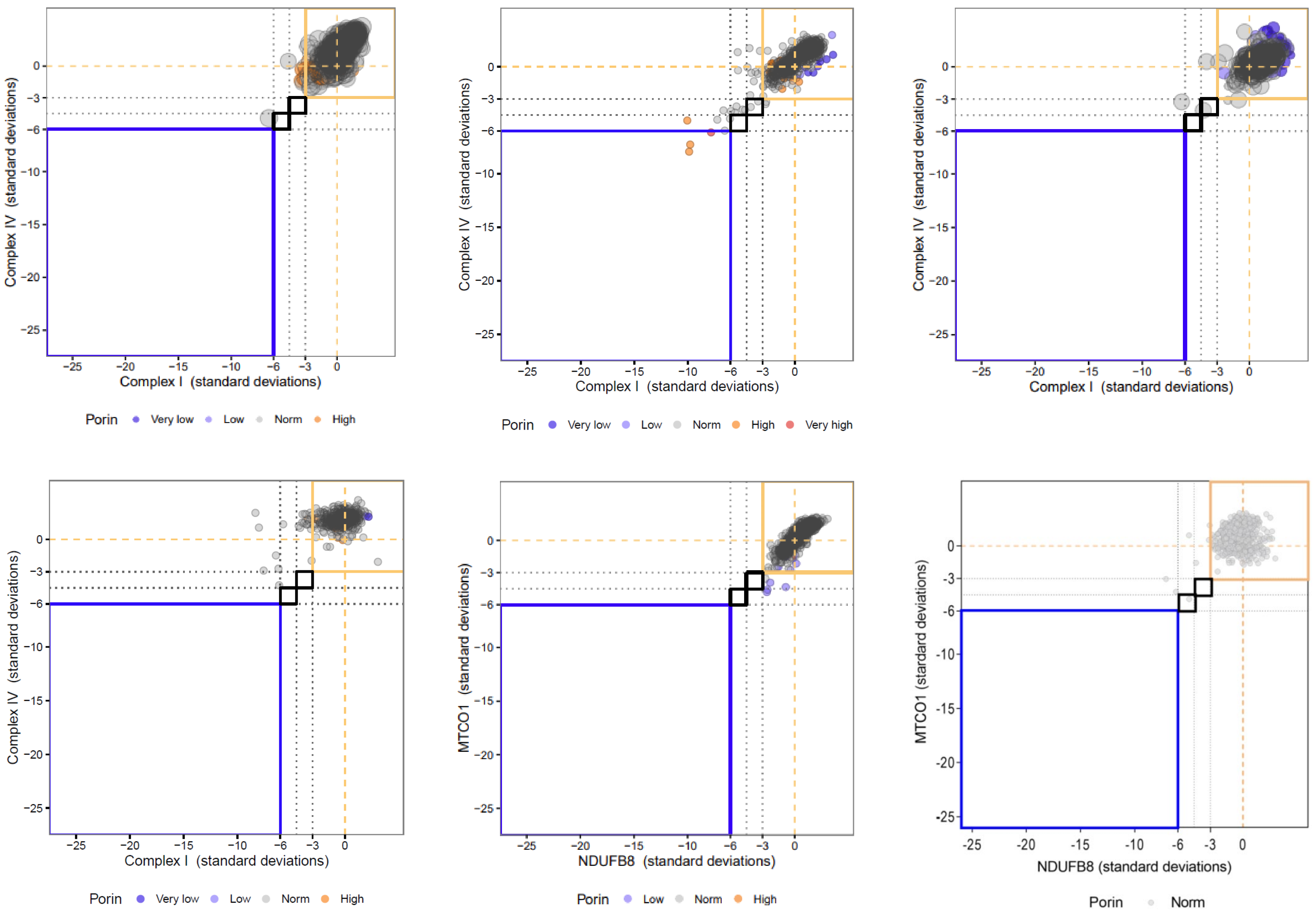

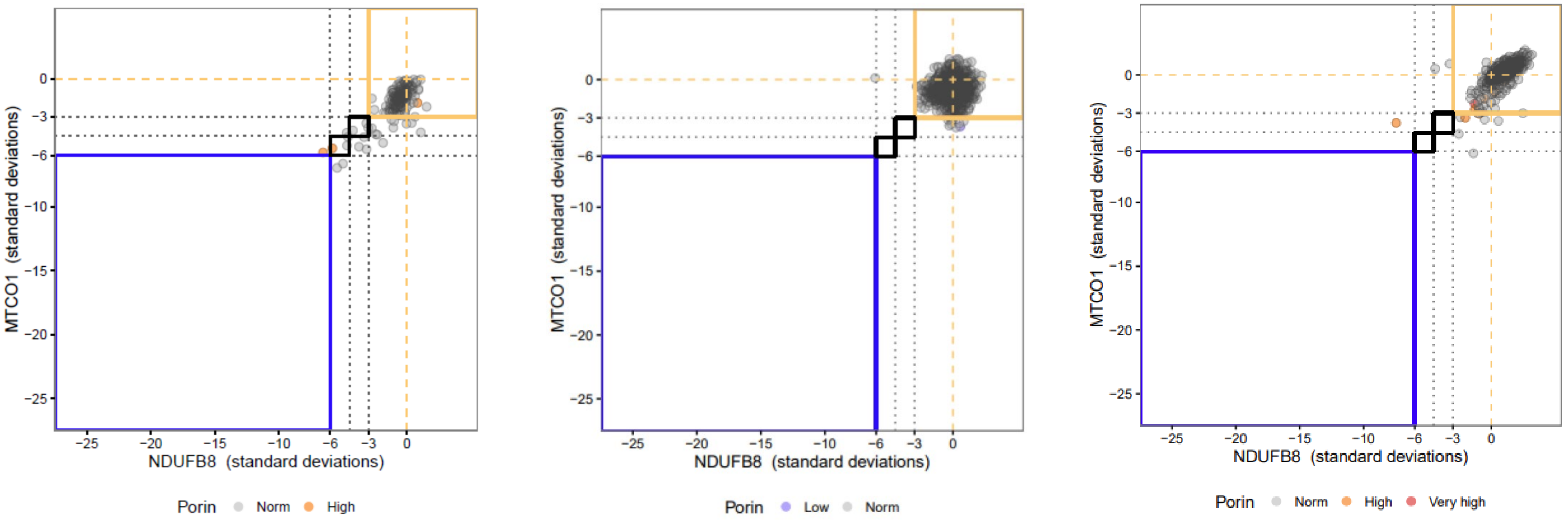

ART-treated PWH

HIV negative

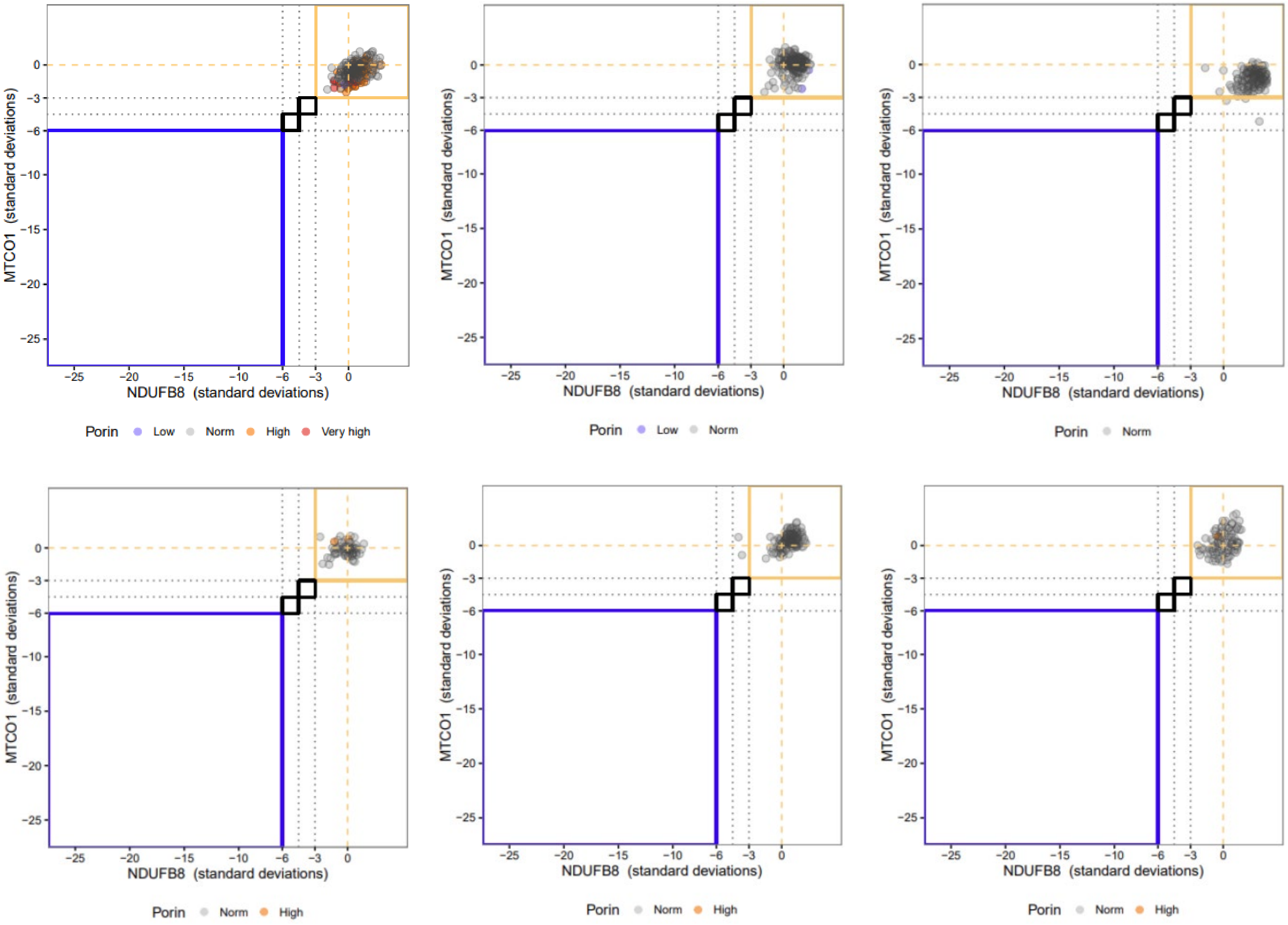

HIV negative

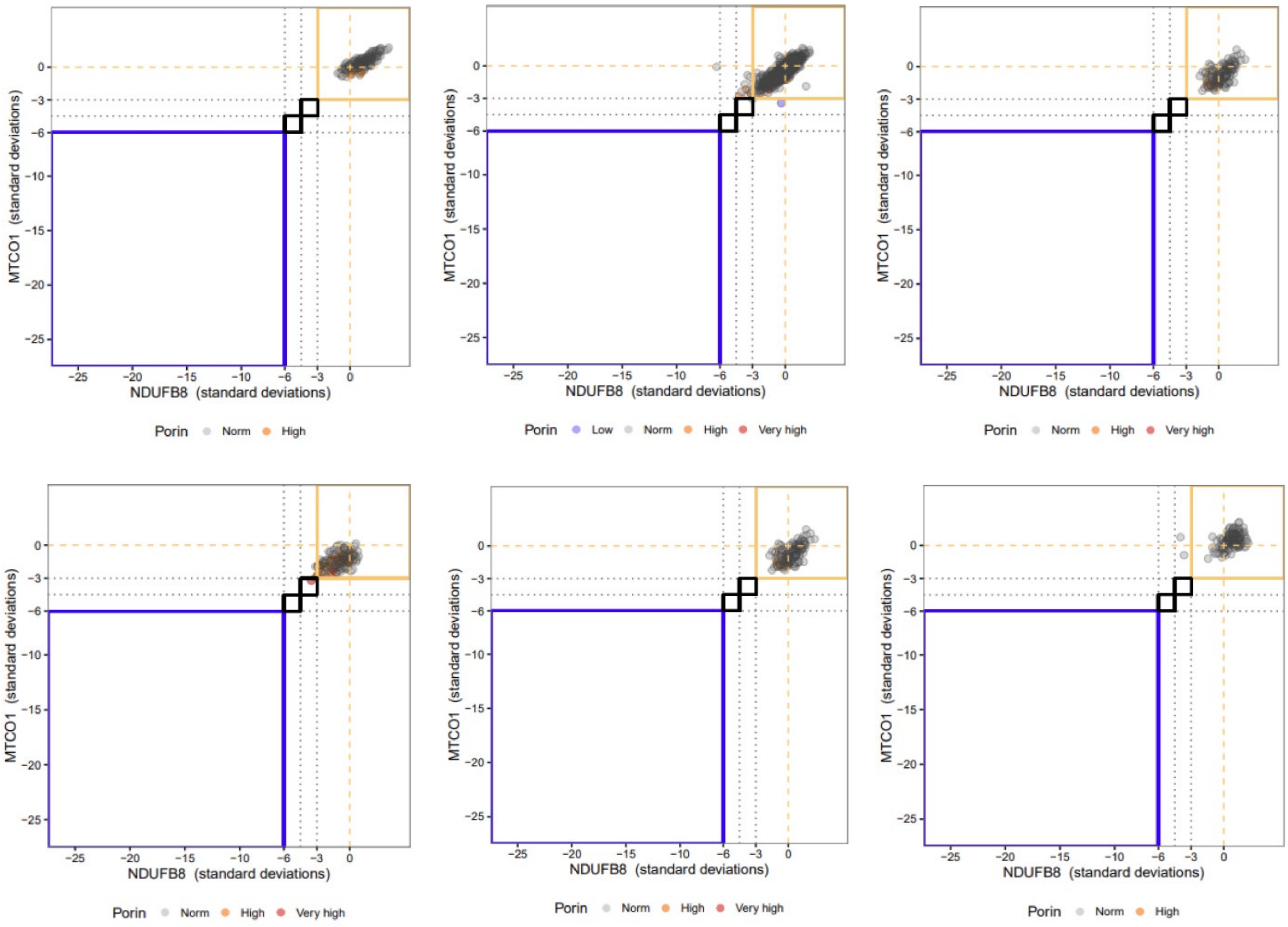

HIV negative

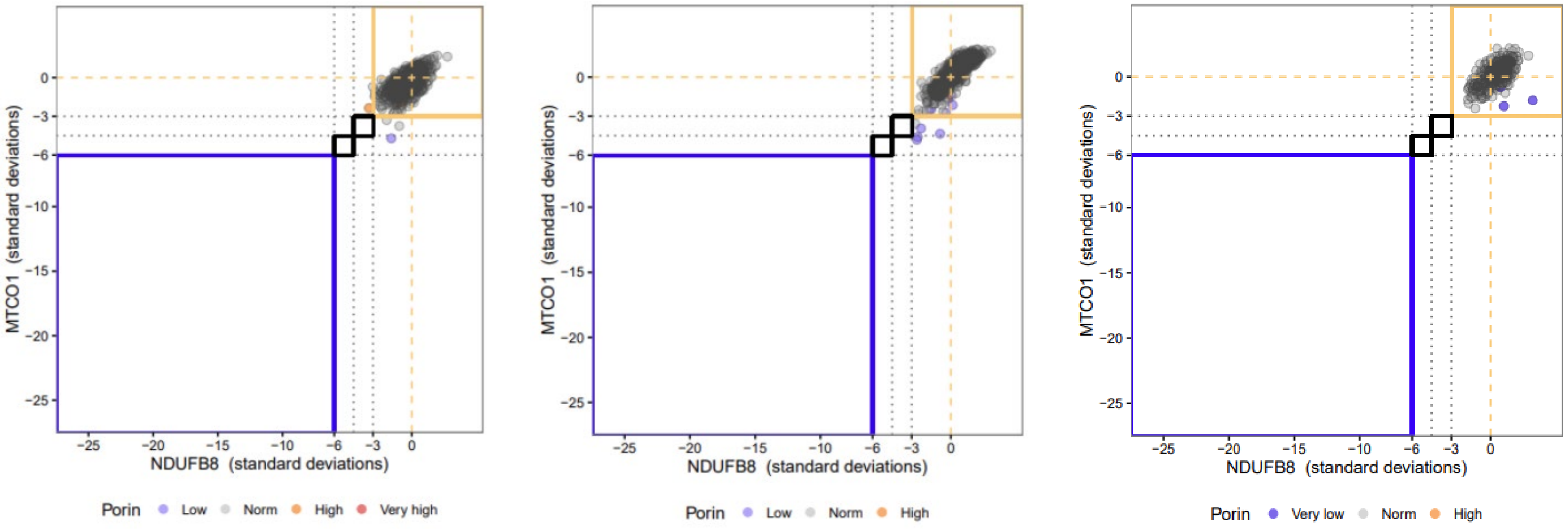

ART-naive PWH

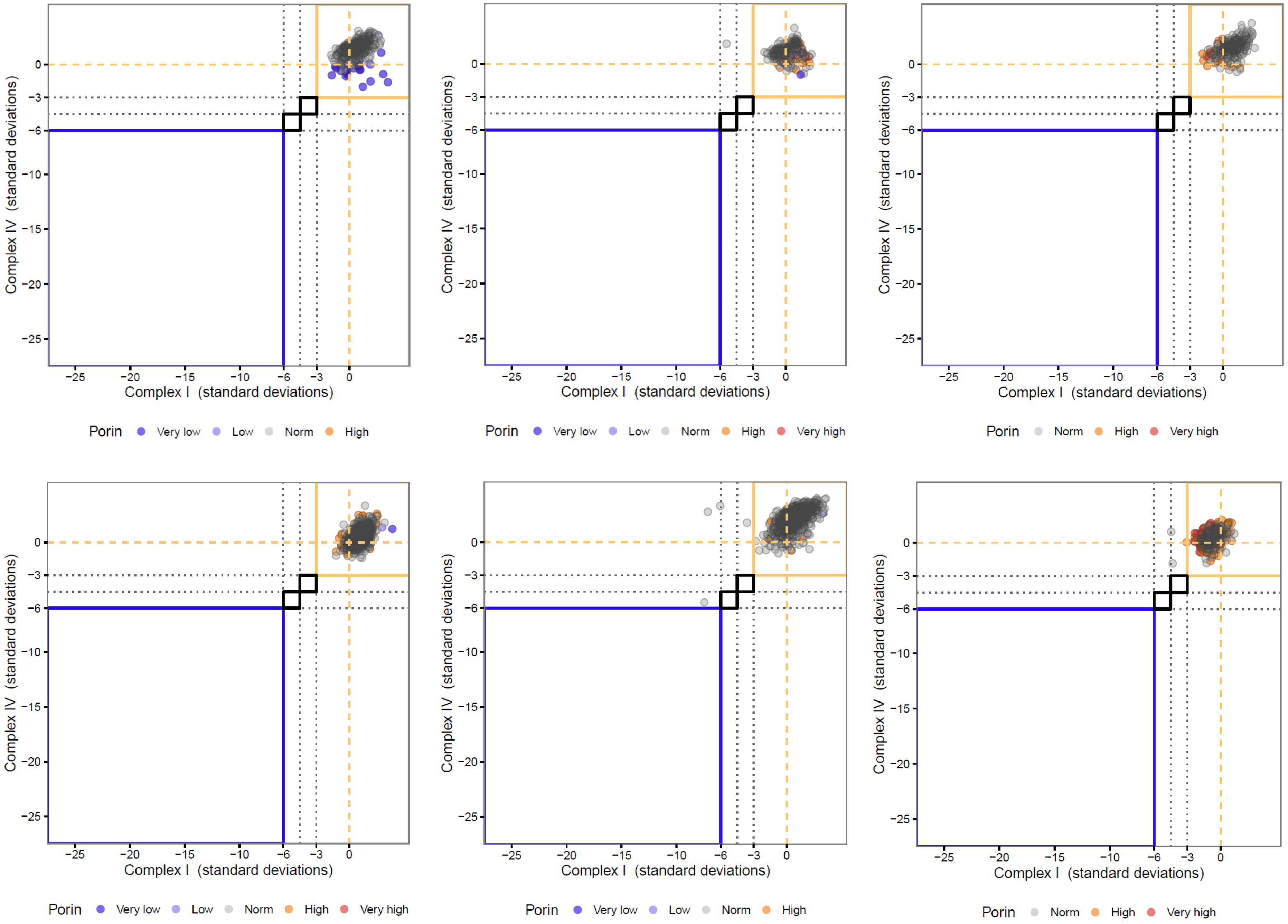

ART-naive PWH

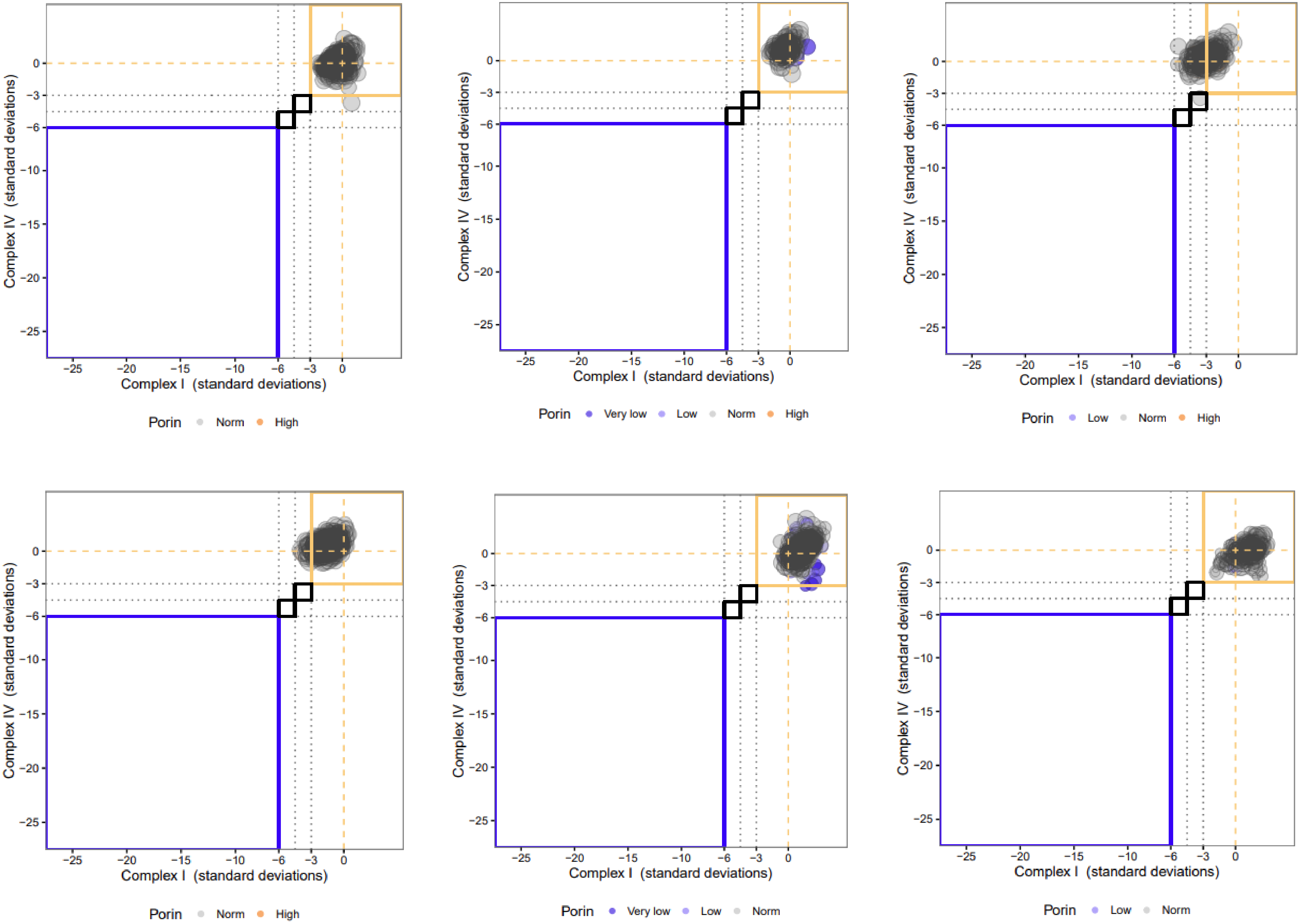

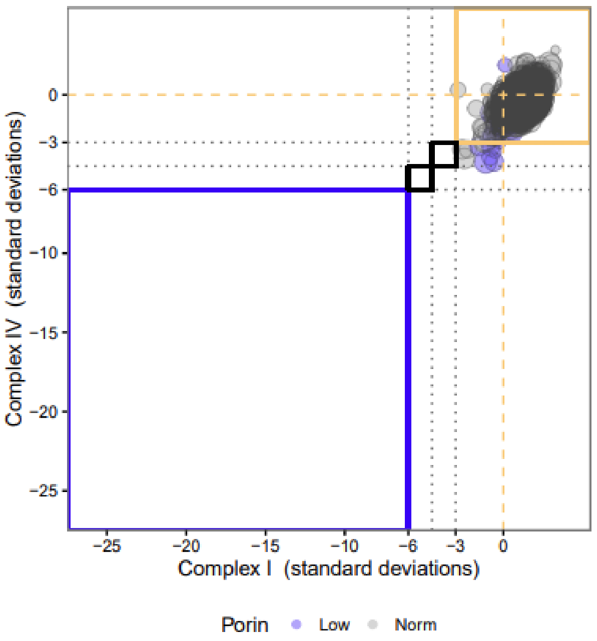

ART-naive PWH
