# Supplementary material for: Skeletal muscle mitochondrial dysfunction in contemporary antiretroviral therapy: a single cell analysis
Source: AIDS. Author manuscript; Available in PMC 2022 Nov 15. (PMC7613767; doi:10.1097/QAD.0000000000003334)
Supplement: Response to reviewers [file EMS150034-supplement-Response_to_reviewers.docx]

**Response to reviewers**

**We would like to thank the reviewer for these helpful suggestions. These have been addressed in full, as follows.**

1. **Methods section
   a. Multiplex immunofluorescence of skeletal muscles (Page 5)
   Most of the data in this paper come from an automated multiplex
   immunofluorescence assay. However, this assay is not described at all. A
   brief description of the assay will help readers understand the results better.**

*Response: A much more detailed description of the underlying principles and rationale of this assay has been added, both in this section and the following one (‘Image analysis’).*

**b. Image analysis
What wavelengths were used for NDUFB8, MTC01, and VDAC1 etc.?
Describe how the z-score was calculated?**

***Response: Wavelengths added. Description of z-score calculation added.***

**c. Single-cell molecular analyses (Page 6)
i. Describe why MT-ND1 and MT-ND4 genes were used? What do they signify? It is important to mention that a region of the ND1 gene is rarely deleted whereas a region of the ND4 gene is deleted in the majority of patients with mitochondrial dysfunction. This is not discussed anywhere in the paper which makes it difficult to understand the results.
ii. What is the control sample used in this assay?
iii. How was the ddCT value calculated?**

***Response: An explanation of the rationale for using qPCR targets in MT-ND1 and MT-ND4 has been added.***

***The control sample used in the qPCR assay has now been explained.***

***The calculation of ddCt has now been explained.*

2. Results section
a. Cohort characteristic
i. Only ART-treated PHW and HIV-negative control groups are mentioned here. ART Naïve and 5 young-healthy individuals should also be mentioned here.**

*Response: Description of the ART-naïve PWH group has now been added.*

*The 5 young healthy control samples are used only as ‘calibrators’ for the immunofluorescence assay. As such they do not form part of the study cohort and are not therefore described here, as that might erroneously imply that they constitute an additional study group. However, the nature and use of these ‘calibrator’ samples is now better described in the methods section.*

**b. Mitochondrial respiratory chain complex deficiency in individual myofibers
i. Figure 1
1. Figure 1 is not discussed in text anywhere. For example, only VDAC1 intensity is discussed in the results section from panel 1A but NDUFB8, MTCO1 and merge panels are not discussed anywhere except figure legend.**

***Response: A more detailed description of Figure 1A has now been added to the results text.***

**2. Figure 1B is not discussed.**

***Response: A brief description of Figure 1B has now been added to the results text.***

**3. Imaging data is the foundation of this work. However, very little imaging data is shown in the manuscript. In Figure 1A, representative immunofluorescence images of mitochondrial mass and respiratory chain complex abundance in individual myofibers are shown for an ART-treated PWH. However, there are no control images. Representative images from HIV-negative control, ART naïve control, and healthy young-individual control samples should be included to make sense of the ART-treated PWH images. Without the control images, figure 1A is not really informative. Ideally, all the images should be provided as supplementary data (as done in references 19 and 20) but at least the control images should be included in Figure 1A.**

***Response: Representative immunofluorescence images from an HIV negative control and an ART-naïve control have now been added to Figure 1A.***

***As also mentioned above, the 5 young healthy controls are actually used as ‘calibrator’ samples for the immunofluorescence assay only and therefore do not form part of the subject groups. They are therefore not included in Figure 1A as this might erroneously imply that they form another subject group.***

***It is not possible to include immunofluorescence images from all subjects as supplementary material as the raw data from this assay are not stored in this format. The images shown in Figure 1A are illustrative images only. The raw data are recorded as optical density values of individual myofibres for each target. Subject-level data can however be provided as supplementary material in the form of 2-D plots (see below).***

**4. Similarly Figure 1B should have a representative 2-D plot of complex I and IV abundance in skeletal muscle sections from control as previously done by other groups for this assay (https://www.nature.com/articles/s41598-017-14623-2#Sec18, See Figure 1A).**

***Response: Representative 2-D plots have now been added for each of the three subject groups.***

***In addition, 2-D plots for all subjects have now been made available as supplementary material.***

**5. What do the box and lines in figure 1B represent?**

***Response: Explanation now added to Figure 1 legend.***

**6. Figure 2A/B/C - what does the dotted line on the x-axis represent?**

***Response: This dotted line was intended to create a separation of the ART-naïve group as it is not directly (statistically) comparable with the other two subject groups (as it is not age-matched – see explanation in the methods section). It is clear from the reviewer’s comment that this dotted line is not helpful and it has therefore been removed.***

**7. Figure 4 - Major arc and minor arc lines on the y-axis need adjustment to match with the legend description.**

***Response: In fact it was the wording of the Figure 4 legend, rather than the axis labelling on the figure that was incorrect. The wording of the Figure 4 legend has now been amended to align with the Figure axis labelling and for better clarity.***

**c. Molecular basis of CI-deficiency in single myofibres
i. Mentions "In keeping with our observations of cellular mitochondrial mass, we saw no evidence of reduced mtDNA content in individual CI-deficient myofibres."
This data is not shown. The other data that shows that mtDNA amount is not reduced comes from the multiplex immunofluorescence assay, however since raw images are not shown for that assay, it becomes very important to show the mtDNA data from the real-time PCR.**

***Response: A new figure panel has been added to show mtDNA content as well as deletions in individual myofibres (now Figure 4A and 4B).***

**ii. QPCR data shows the presence of mtDNA deletions. It will be good to perform an analysis of Complex I assembly by BN-PAGE to confirm a decrease in fully-assembled CI. This is not essential for this study though.**

***Response: This is an interesting point that the reviewer makes. It is not possible to add this analysis to the present study, but we have added it as suggested future work in the discussion (at end of the penultimate paragraph).***
